# A deep learning model for detecting mental illness from user content on social media

**DOI:** 10.1038/s41598-020-68764-y

**Published:** 2020-07-16

**Authors:** Jina Kim, Jieon Lee, Eunil Park, Jinyoung Han

**Affiliations:** 1 Department of Interaction Science, Sungkyunkwan University, Seoul, 03063 Republic of Korea; 2 School of Computer Science, Carnegie Mellon University, Pittsburgh, PA 15213 USA; 3 Department of Applied Artificial Intelligence, Sungkyunkwan University, Seoul, 03063 Republic of Korea

**Keywords:** Health care, Engineering

## Abstract

Users of social media often share their feelings or emotional states through their posts. In this study, we developed a deep learning model to identify a user’s mental state based on his/her posting information. To this end, we collected posts from mental health communities in *Reddit*. By analyzing and learning posting information written by users, our proposed model could accurately identify whether a user’s post belongs to a specific mental disorder, including depression, anxiety, bipolar, borderline personality disorder, schizophrenia, and autism. We believe our model can help identify potential sufferers with mental illness based on their posts. This study further discusses the implication of our proposed model, which can serve as a supplementary tool for monitoring mental health states of individuals who frequently use social media.

## Introduction

Social media is a popular space for expressing users’ feelings^1,2^. Through diverse social media or online social health communities, users often are likely to present their mental problems or illness with anonymity^3^. Such online health communities can be a network for expressing sympathy by communicating with others who have similar symptoms^4^. In addition, users often try to obtain health information related to their symptoms on social media as an attempt to diagnose themselves^5,6^.

With this trend, several scholars have analyzed user-generated content on social media for observing users’ emotional state or mental illness, including depression, anxiety, or schizophrenia^3,6–10^. A recent study collected *Twitter* posts of users who reportedly had been diagnosed as depression^7^, analyzed the linguistic and emotional characteristics of the collected posts using the Linguistic Inquiry and Word Count (LIWC)^11^, and tracked their social engagement changes on *Twitter*. Another study attempted to predict users’ postpartum depression on *Facebook*, based on their posts and comments, and used specialized psychometric instruments to evaluate the level of postpartum depression between pre- and post-natal periods^12^. In addition, Reece et al.^13^ used image data to detect users’ depression on social network services. After collecting photos from *Instagram* uploaded by users, both face detection and colorimetric analysis were applied. To detect users’ anxiety disorders, prior research collected user data from *Reddit* and showed that *N*-gram language modeling and vector embedding procedures with topic analysis of users’ posts are efficient in finding potential users with anxiety disorders^3^.

Several previous studies revealed that social media data is useful in observing or detecting users’ emotions or potential mental problems. This study goes one step further; by collecting various mental-health-related data from social media, we aim at developing a deep learning model that can identify a user’s mental disorder, including depression, anxiety, bipolar, borderline personality disorder (BPD), schizophrenia, and autism. To this end, we collected users’ posts from *Reddit*, a popular social media that includes numerous mental-health-related communities (or so-called ‘subreddits’), such as r/depression, r/bipolar, and r/schizophrenia^8^. As our aim is to identify whether a user suffers from a mental illness, such as depression and anxiety^14^, we collected data from the six subreddits: r/depression, r/Anxiety, r/bipolar, r/BPD, r/schizophrenia, and r/autism. Note that we employed the mental-health-related subreddits identified in prior work^10^. More specifically, among the popular 83 subreddits, 6 subreddits were identified as mental-health-related ones by a statistical approach like a semi-supervised method as well as an assessment procedure by experts^10^. Each identified subreddit is associated with a specific mental condition, e.g., r/depression is associated with the depression condition.

By collecting and analyzing user’s posts uploaded in multiple mental-health-related subreddits in *Reddit*, we investigated whether specific posts of the user can be classified as relevant types of mental disorder. People who suffer from specific mental disorders may not know their most accurate diagnosis; for example, people with bipolar disorder can have a hard time distinguishing bipolar from depression since the symptom of both is similar^6,15^, or even it is strenuous to initially diagnose bipolar disorder^16^. We assumed that users attempt to search for mental health information on social media with general keywords, such as ‘mental health’, ‘mental illness’, or ‘mental status’, as if they reach out for help by opening up general stories about them at an early stage. Subsequently, many users are likely to communicate with other users in one of the general health-related channels in *Reddit* (e.g., r/mentalhealth) in the beginning, but often fails to recognize their accurate problems. Therefore, we attempt to detect users’ potential mental disorders by their posts on social media. This study seeks to address the following research question.

- **Research question**: Can we identify whether a user’s post belongs to mental illnesses on social media?

## Study method

### Data collection

We collected post data from the following six mental-health-related subreddits, each of which is reported to be associated with a specific disorder^10^: r/depression, r/Anxiety, r/bipolar, r/BPD, r/schizophrenia, and r/autism. In addition, we further collected post data from the most popular health-related subreddit^17^, r/mentalhealth, to analyze posts with general health information. From each subreddit, we collected all the user IDs who had at least one post related to the mental health. Along with user IDs, we also collected titles and posts using the *PushshiftAPI*^18^.

Note that all the user information is anonymized, hence no personally identifiable information was not included; we followed all the anonymization process guided by the Sungkyunkwan University Institutional Review Board (IRB). Overall, the current study collected information from 248,537 users, who wrote 633,385 posts in the seven subreddits from January 2017 to December 2018. Table 1 summarizes the information of collected data.

**Table 1:** A summary of the collected data from *Reddit* (http://reddit.com/).

Channel	# of users	# of posts	Description
r/mentalhealth	27,177	39,373	The Mental Health subreddit is the central forum to discuss, vent, support and share information about mental health, illness and wellness
r/depression	136,506	258,496	Peer support for anyone struggling with depression, the mental illness
r/Anxiety	49,735	86,243	Discussion and support for sufferers and loved ones of any anxiety disorder
r/bipolar	14,372	41,493	A safe haven for bipolar related issues. We are a community here not just a help page. Be a part of something that cares about who you are
r/BPD	13,913	38,216	A place for those who have BPD (Borderline Personality Disorder) (also known as EUPD [Emotionally Unstable Personality Disorder])–, their family members and friends, and anyone else who is interested in learning more about the disorder
r/schizophrenia	5,392	17,506	Welcome! This is a community meant for a discussion of schizophrenia spectrum disorders, and related issues. Feel free to post, discuss, or just lurk. There is no judgement in this place: we are here for each other. Please refrain from self-diagnosis, diagnosing others, or advising specific medical treatments
r/autism	4,754	7,142	No description

### Data pre-processing procedure

**Figure 1:**
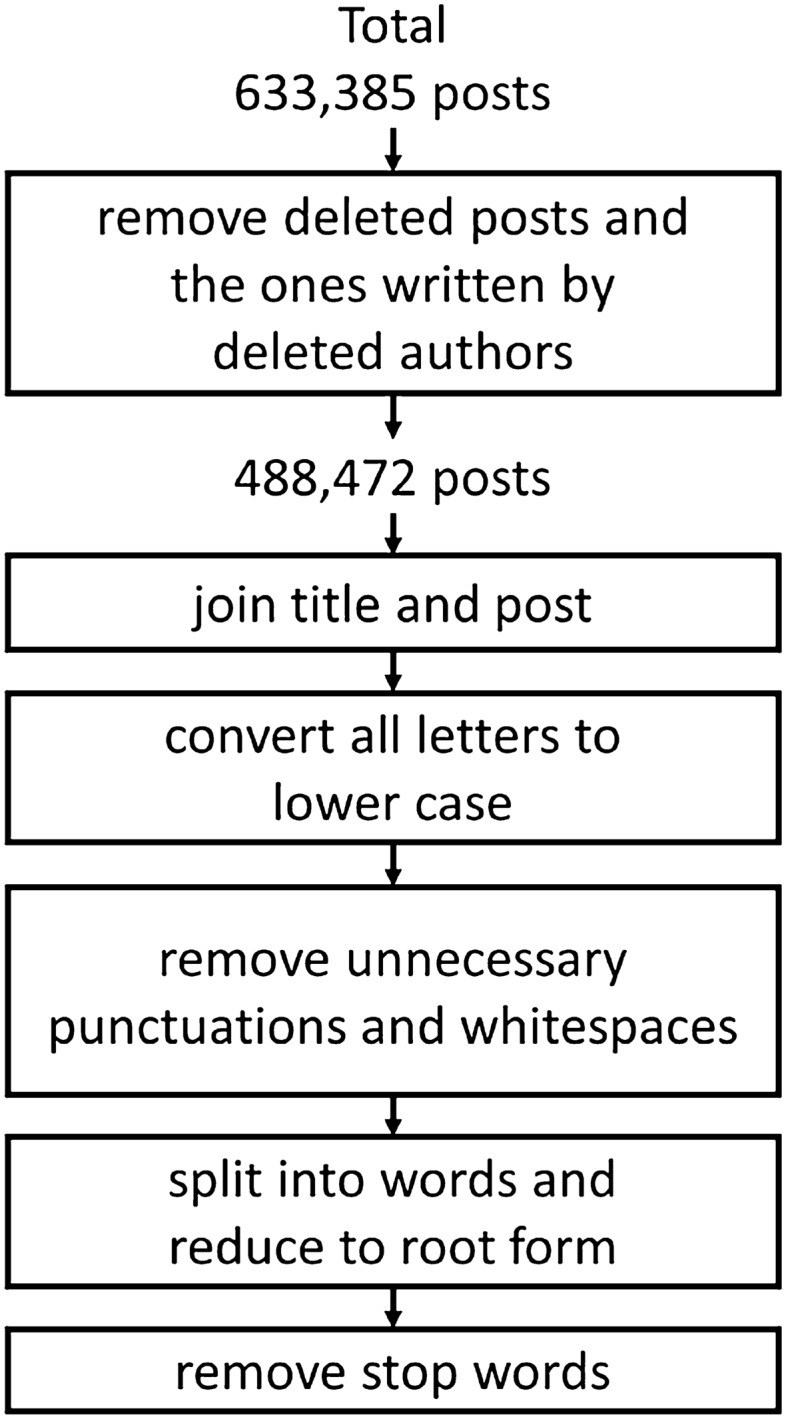
A data pre-processing procedure.

The data pre-processing procedure for the collected post data is presented in Fig. 1. After collecting the data, each title was combined with its corresponding post. We removed unnecessary punctuation marks and white spaces for each post. Then, we used the natural language toolkit (NLTK) implemented in Python to tokenize users’ posts and filter frequently employed words (stop words). Porter Stemmer, a tool used to define a series of guidelines for exploring word meaning and source, was employed on the tokenized words, to convert a word to its root meaning and to decrease the number of word corpus. After this procedure, data from 228,060 users with 488,472 posts in total were employed for the analysis.

### Classification models

We developed six binary classification models, each of which categorizes a user’ specific post into one of the following subreddits: r/depression, r/Anxiety, r/bipolar, r/BPD, r/schizophrenia, and r/autism. Our conjecture is that a user who suffers from a specific mental problem writes a post on the corresponding subreddit that deals with the problem. A user can write posts across multiple subreddits if he/she suffers from multiple mental health problems, e.g., a user suffering from both depression and anxiety. However, if the model is trained with the posts of users who have multiple symptoms like a prior study^10^, the classification model may suffer from noisy data. Therefore, we developed six independent binary classification models for each symptom to improve the performance. By developing six independent models for each mental disorder, each of which uses data where users suffer from only one particular mental problem, we were able to accurately identify a user’s potential mental state. For example, to develop a model for detecting depression, we labeled the posts written by users who upload posts only in the r/depression as the *depression* class; the opposite class is referred to as the *non-depression* class. To address a class-imbalance issue for the collected data, we applied the synthetic minority over-sampling technique (SMOTE) algorithm^19^.

We divided our dataset into training (80%) and testing (20%) sets. Then, XGBoost and convolutional neural network (CNN) were employed. Morover, we excluded the posts of users who wrote posts across multiple subreddits in learning phase.

**Figure 2:**
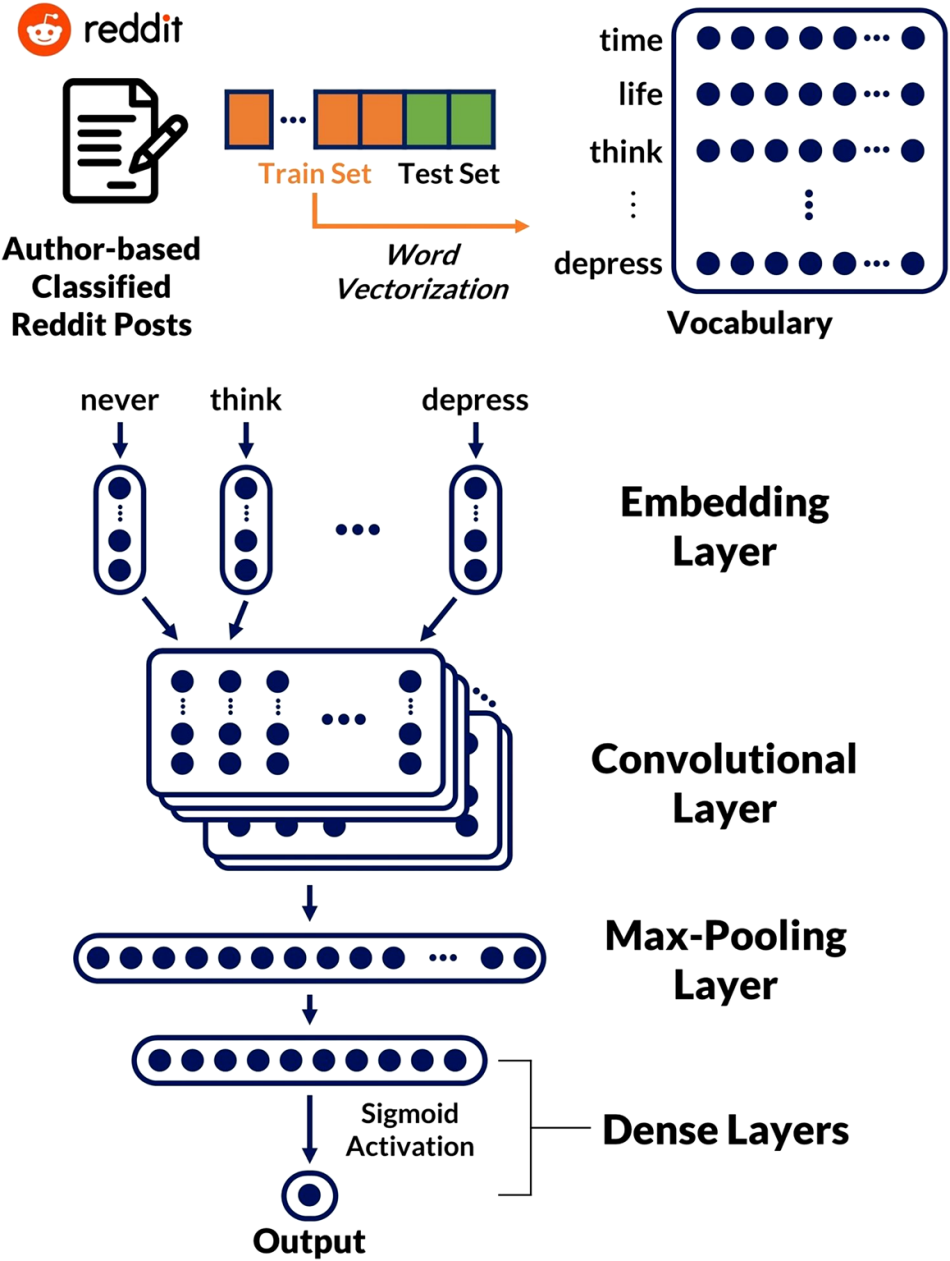
An architecture of the proposed CNN-based classification model.

To quantitatively represent each post, we converted the words in the training set to numerical representations (Fig. 2). For the XGBoost classifier, we used the *TF-IDF* vectorizer in the sckit-learn package^20^ to convert words into *n*-dimensional vectors. In the case of the CNN classifier, we applied word-embedding procedures from the pre-processed texts using the word2vec API of Python Package, *Gensim*^21^. The word vectors were pre-trained with the training dataset collected for the current study with continuous bag-of-words representation (CBOW) models, while the size of window was set to five. Note that by using the pre-trained word2vec model for representing each post for each subreddit, a language style used by users who write posts in a subreddit can be trained for the specific subreddit.

An overview of the proposed CNN-based model is presented in Fig. 2. The model architecture is organized by the sequence of layers that includes an embedding layer, convolutional layer, max-pooling layer, dense layers, and the output. Fig. 2 illustrates how a post is trained in the given model. The first layer of the model is an embedding layer that represents the word embeddings of a pre-processed post with 20 dimensions, and its weight is initialized by the pre-trained word2vec. Second, a convolutional layer with input of word vectors has 128 filters, and each filter size is five. In addition, we applied a dropout rate of 0.25 to prevent over-fitting issues. The next layer is a max-pooling layer, which takes the maximum values within the CNN filters, and its dimension is 128. The output of the max-pooling layer is passed through two fully connected (dense) layers, and the final output is the probability of the classification through the sigmoid activation function, which ranges from 0 to 1. For training the neural network, we used both the binary cross-entropy loss function and Adam optimizer^22^, with a learning rate of 0.001. Our model was trained through 50 epochs and the batch size was set to 64.

### Ethics declarations

This study was approved by the Ethical Committee and Institutional Review Board of the Department of Applied Artificial Intelligence, Sungkyunkwan University (#H1AAI2020).

## Results

Four evaluation metrics were employed to validate the performance of the models: accuracy (Eq. 1), precision (Eq. 2), recall (Eq. 3), and F1-score (Eq. 4). *TP*, *FN*, *TN*, and *FP* represent true positive, false negative, true negative, and false positive, respectively.

1 $$\begin{aligned} accuracy= \frac{TP + TN}{TP + FN + TN + FP} \end{aligned}$$

2 $$\begin{aligned} precision= \frac{TP}{TP + FP} \end{aligned}$$

3 $$\begin{aligned} recall= \frac{TP}{TP + FN} \end{aligned}$$

4 $$\begin{aligned} F1\text{-}score= \frac{2 \times precision \times recall}{precision + recall}. \end{aligned}$$

Table 2 summarizes the performance of the six binary classification models. Among the six different subreddits, r/autism showed the highest accuracy (96.96%) in the CNN, but had the lowest F1-score on the *autism* class (XGBoost: 38.31%, CNN: 48.73%), which is due to the class imbalance problem. Overall, CNN models showed higher accuracy than XGBoost models across all the subreddits. One of the most class-balanced subreddits, r/depression, showed the highest performance scores in terms of precision (89.10%), recall (71.75%), and F1-score (79.49%) for the *depression* class. Three other subreddits, r/Anxiety, r/bipolar, and r/BPD, also showed high accuracy with CNN models, 77.81%, 90.20%, and 90.49%, respectively, and their F1-scores in identifying mental illnesses ranged from forties to fifties (%), which are relatively lower than those with the class-balanced channels. In summary, our proposed model can accurately detect potential users who may have psychological disorders. We believe collecting more data may resolve the imbalanced data problem, resulting in a better performance.

**Table 2:** Model evaluation of XGBoost and Convolutional Neural Network.

Channel	Class	XGBoost		CNN			
		F1-Score	Accuracy	Precision	Recall	F1-Score	Accuracy (%)
r/depression	Depression	58.02	71.69	89.10	71.75	79.49	75.13
	Non-depression	78.65		58.66	82.04	68.41	
r/Anxiety	Anxiety	55.92	70.41	87.54	41.44	56.25	77.81
	Non-anxiety	77.73		75.92	96.91	85.14	
r/bipolar	Bipolar	53.59	85.53	87.22	38.02	52.95	90.20
	Non-bipolar	91.43		90.40	99.05	94.53	
r/BPD	BPD	46.43	85.14	91.84	32.69	48.21	90.49
	Non-BPD	91.37		90.42	99.54	94.76	
r/schizophrenia	Schizophrenia	40.97	86.72	81.16	24.87	38.07	94.33
	Non-schizophrenia	92.52		94.62	99.56	97.03	
r/autism	Autism	38.31	94.91	48.08	49.39	48.73	96.96
	Non-autism	97.35		98.48	98.40	98.44	

## Discussion

Detecting mental illness problems in early stages and providing appropriate solutions can help potential mental disorder sufferers^23^. By collecting and analyzing data from mental-health-related subreddits in *Reddit* that focus on mental disorder issues, we introduced a deep learning model with natural language processing methods to identify the users with potential mental illness based on their posts. We believe that our method can open up a new research era where online social media can play a role as an efficient source for identifying potential mental illness based on users’ specific posts^24^. However, a majority of people who may have mental illness are still in social blind spots and lacks appropriate treatment due to several reasons such as difficulty in revealing their status to someone in person or having difficulties in physically accessing the clinics^23^.

Based on the lessons learned, the following implications are presented. First, deep learning approaches with appropriate natural language processing methods can be used to detect users’ potential mental illnesses by their posts. With the employment of easily accessible social media data, the approaches used in this study can be adopted to alert the users who may be suffered from specific mental disorders before they visit counseling centers. Second, this study provides notable evidence supporting the possibility of utilizing online platforms that can help people in need of mental treatment. Specifically, for example, online platform service providers may ask a user’s consent first to access one’s account and if agreed, can provide the probabilities of each mental disorder predicted through our validated models based on the user’s posts. Lastly, the current study suggests detecting mental illness in social media can be a prominent research area in the future. The findings of the current study reveal the potential for social media platforms that can play a role in providing a space to interact with others who are suffered by mental disorder.

However, there are a few limitations in this study. The current study did not consider several factors (e.g., socio-demographic and regional differences) that could affect the classification models. These factors can be considered in future research, which can improve the quality or accuracy of the deep learning models. In addition, we collected the data from the public social media, *Reddit*, which may be different from the personal feed of social network services in expressing users’ emotions. We did not conduct additional validation procedures of our model with another independent dataset as mentioned above, which would need to be further investigated. Although postings in online social media could not explicitly tell the symptoms compared to posts in users’ personal pages that may say they are diagnosed with clinical mental illnesses, online social media have a potential to be used to identify mental disorder sufferers because they share their symptoms relatively accurately under the semi-anonymity system. Also, we trained our model on a specific mental state to directly classify the symptom and provide the predicted probabilities for each symptom. In this way, we could not accurately measure the co-morbid mental illness status, which is left for future work.

In future study, we could adopt an ensemble approach with our multiple binary classification models, which can be utilized to identify the real-world mental conditions, such as co-morbid illness. We also plan to validate our proposed model in posts of users who may have uncertain mental disease in other social network services such as *Facebook* or *Twitter*. In addition, a time-series user-level analysis that tracks a users’ longitudinal behavior pattern can help to develop a user-level detection model for mental illness using a recurrent neural network.

## Data Availability

The collected data in this paper can be achieved at https://jina-kim.github.io/dataset/20srep-mental. Other information used in this study can be accessed from the corresponding author with the reasonable request.
